# Anamnestic Immune Response to Dengue and Decreased Severity of Yellow Fever

**DOI:** 10.4103/0974-777X.56257

**Published:** 2009

**Authors:** Ricardo O Izurieta, Maurizio Macaluso, Douglas M Watts, Robert B Tesh, Bolivar Guerra, Ligia M Cruz, Sagar Galwankar, Sten H Vermund

**Affiliations:** *Department of Global Health, College of Public Health, University of South Florida, Tampa, Florida, USA*; 1 *Department of Epidemiology and International Health, School of Public Health, University of Alabama at Birmingham, Birmingham, Alabama, USA*; 2 *Department of Pathology, Center for Tropical Diseases, University of Texas Medical Branch, Galveston, Texas, USA*; 3 *Ecuadorian Armed Forces Hospital HG-1, Quito, Ecuador, USA*; 4 *Department of Global Health, College of Public Health, University of South Florida, Tampa, Florida, USA*; 5 *Institute for Global Health, School of Medicine, Vanderbilt University, USA*

**Keywords:** Antibody, Cross-protection, Dengue, Immunology, Yellow fever

## Abstract

A protective immunity against yellow fever, from cross-reactive dengue antibodies, has been hypothesized as an explanation for the absence of yellow fever in Southern Asia where dengue immunity is almost universal. This study evaluates the association between protective immunity from cross-reactive dengue antibodies with yellow fever infection and severity of the disease. The study population consisted of military personnel of a jungle garrison and its detachments located in the Ecuadorian Amazonian rainforest. The cross-sectional study employed interviews as well as seroepidemiological methods. Humoral immune response to yellow fever, Mayaro, Venezuelan equine encephalitis, Oropouche, and dengue 2 infections was assessed by evaluating IgM and IgG specific antibodies. Log-linear regression analysis was used to evaluate age and presence of antibodies, against dengue type 2 virus, as predictors of yellow fever infection or severe disease. During the seroepidemiological survey, presence of dengue antibodies among yellow fever cases were observed in 77.3% cases from the coastal region, where dengue is endemic, 14.3% cases from the Amazon and 16.7 % cases from the Andean region. Dengue cross-reactive antibodies were not significantly associated with yellow fever infection but significantly associated with severity of the disease. The findings of this study suggest that previous exposure to dengue infection may have induced an anamnestic immune response that did not prevent yellow fever infection but greatly reduced the severity of the disease.

## INTRODUCTION

A protective immunity against yellow fever, from cross-reactive dengue antibodies, has been hypothesized as an explanation for the absence of yellow fever in Southern Asia where dengue immunity is almost universal. Similarly, residents of central Africa and the entire eastern coast of Africa have traditionally escaped the frequent yellow fever outbreaks on the West Coast.[1]

In the 19^th^ century it was observed that indentured laborers from India and British troops, who had served in India, were less susceptible to yellow fever during the epidemic that occurred in America.[2] After the emancipation of slaves in the British West Indies, in 1788, hundreds of thousands of indentured laborers were brought from India to the British colonies of Trinidad and Guiana. These Indian workers, like the resident populations, were little affected by the outbreak of yellow fever which principally attacked European sailors.

Three theories have been proposed to explain the absence of yellow fever in Asia: (1) Protective immunity from dengue and other flavivirus cross-reactive antibodies; (2) Relatively low competence of Asian strains of *Aedes aegypti* mosquitoes to transmit yellow fever virus; and (3) Demographic and geographic obstacles to the spread of yellow fever virus which tend to occur in relatively remote areas.[3]

The well-documented serological cross-reactions observed over years among flaviviruses strongly suggest the presence of common antigens for members of this group. It is conceivable that a prior immunological experience with one member of this group might facilitate a secondary “booster” upon subsequent exposure to a different, but related virus. Primary yellow fever infection is followed by the appearance of a specific antibody response that is identifiable by most methods. In contrast, people with previous flavivirus immune experience can develop a rapid and broadly cross-reactive response. In these cases, response to previous flavivirus infections may be stronger than the yellow fever-specific response, illustrating the “doctrine of the original antigenic sin”.[4]

Activation of memory T lymphocytes during a secondary flavivirus infection and shared common antigenic determinants generally related to the E protein have been observed in experimental studies.[5,6] Therefore, it is plausible that both flavivirus cross-reactive antibodies and T cells may prevent or aid in the recovery from other flavivirus infection. There is epidemiological evidence, though not solid, to confirm that immunity to one flavivirus provides protection against infection by other flavivirus. Shu-Yuan Xiao *et al*. tested the hypothesis that prior infection with heterologous flavivirus protects against severe or fatal yellow fever using a hamster model of the disease; their results not only support the hypothesis but also identify a reduced level of viremia and less severe liver function damage in the animal model.[7]

This study analyzes the hypothesized protective effect of cross-reactive dengue antibodies, against yellow fever, using information from a jungle yellow fever outbreak that attacked military personnel with previous dengue exposure.

## MATERIALS AND METHODS

*Study population:* The jungle garrison under study consisted of 348 subjects detached in one main post, three detachments and five outposts, all located in the Amazonian rainforest near the Peruvian border. From the total population 10 subjects were not included in the immunological study because of unavailability of sera (seven) and three fatal cases. This geographic area, situated at 100 meters above sea level, is classified as humid tropical forest. The province has approximately 57,000 inhabitants, with an average population density of 1.92 inhabitants per square kilometer.

*Study procedures:* A cross-sectional seroepidemiological survey was conducted in the study population. After providing written informed consent, study subjects participated in a questionnaire interview concerning demographic variables, medical history and potential risk factors. In a few cases, because of the severity of patients' condition, recent medical history data was obtained at the Military Hospital No 1 of Quito (HG-1) rather than from direct patient interviews.

Blood samples were processed immediately after collection, and sera were stored frozen at minus 20°C until shipped. Serum samples were transported on dry ice to the U.S. Naval Medical Research Institute Detachment (NAMRID) in Lima, Peru for viral isolation and serologic testing. The Ecuadorian Armed Forces and the U.S. Navy Guidelines for the use of human subjects were followed. All procedures followed international guidelines for research on human subjects and were supervised by the Ecuadorian National Council against hemorragic fevers complemented by health officers representing the Ecuadorian Armed Forces and the Ministry of Public Health. *Serology*. Confluent monolayers of LLCMK_2 or_ Vero cells were infected with prototype dengue (DEN)-1, Oropuche (ORO) Peru 1992,[8] yellow fever 17D, Venezuelan equine encephalitis (VEE) subtype I-AB virus, (Trinidad donkey) and Mayaro (MAY) TR467 strains. The resulting supernatant viral antigens were used to test sera for IgM antibody by a capture enzyme linked immunosorbent essay (ELISA),[9] and for preparing lysate viral antigens for performing IgG antibody ELISA.[10,11] A capture of ELISA using goat anti-human IgM (Tago, Camarillo, CA., U.S.A.) bound to 96-well Limbro microtiter plates was used to test for IgM antibodies.[9] An indirect ELISA employing viral infected or uninfected cell lysates bound to 96 well microtiter plates was used to test serum for IgG antibody.[12] Sera were tested initially at 1:100 dilution, and reactive samples were further tested at a 1:200 through 1:12,800 dilutions to determine the antibody endpoint titer. Virus specific IgM and IgG antibodies- positive and negative controls (run at a 1:100 dilution) were included in each test to validate the results. A horseradish peroxidase (HRP, Kirkegard and Perry, Gaitherburg, MD, U.S.A.) conjugated anti-mouse IgG, and an enzyme substrate 2,2 ‘-azino di(3-ethyl-benzthiazoline) sulfonate (ABTS) were used to detect IgM antibody. HRP conjugated goat anti-human IgG and ABTS were used to detect IgG antibody. The absorbance was read with a spectrophotometer at 414 nm wavelength. The absorbance values for the mock antigens were subtracted from those of the viral antigen to yield corrected absorbance values. Serum dilutions with corrected absorbance values greater than the reference cut-off value, estimated as the mean absorbance of 10 antibody-negative serum samples plus 3 standard deviations, were considered antibody positive. Samples with antibody titers of 1:200 or greater were classified as positive. All antibody titers were expressed as the reciprocal of the highest dilution yielding a positive result.

The laboratory procedures reported herein were conducted according to the principles set forth in U.S. Federal guidelines (Guide for the Care Use of Laboratory Animals. Institute of Laboratory Animal Resources, National Research Council, DHHS, Publication No. (NHI) 86-23, 1985).

The specificity of antibody reactivity was determined by a standard plaque reduction neutralization test (PRNT) employing Vero cell Cultures. A sample of yellow fever ELISA IgG antibody positive sera was diluted 1:10 in Eagle's minimum essential medium (EMEM) supplemented with 2% fetal calf serum (FCS) heat treated at 56°C for 30 minutes. After each dilution was incubated with approximately 100 plaque-forming units (PFU) of virus, aliquots of the mixtures were tested in Vero cell cultures propagated in 25 cm^2^ flasks. Sera that reduced the viral PFU dose by 50% or greater were considered positive for yellow fever antibody.

### Virus isolation and identification

Serum samples obtained from the participants were diluted 1:5 in EMEM supplemented withfive per cent FCS, 200 μg/ml of streptomycin and 200 units/ml of penicillin. Aliquots of each diluted serum sample were assayed for virus by the newborn mouse and Vero cell culture assays.[13]

Aliquots of the original serum that yielded suspected isolates were inoculated intracerebrally into one to three-day-old outbred mice in attempts to re isolate the viruses. Mice were observed daily. All mice which showed signs of illness or died were stored at minus 70°C until the brain tissue was extracted for viral identification studies.

The reference virus used for preliminary identification of viral isolates by the standard indirect immunofluorescence (IFA) technique included VEE, subtype I-AB 69Z1; MAY TR467; ORO TR9760 and yellow fever 17D strain, (American Type Culture Collection (ATCC), Rockville, MD, U.S.A.). Viral antigens and reference virus stocks were prepared from infected Vero cell cultures according to standard procedures. Aliquots of 20 μl of antigen and uninfected control cells were added to microscope slides, air dried, and fixed in cold acetone for 30 minutes. Slides were air dried at room temperature and stored at minus 70°C until used for preliminary identification of viral isolates.

Reference hyperimmune mouse ascitic fluid (MAF) used to perform the IFA was obtained from the National Institute of Allergy and Infectious Diseases (NIAID). These included Alphavirus, Flavivirus and Simbu virus grouping reagents, Western equine encephalitis (WEE), Eastern equine encephalitis, ORO and normal MAF R143. Monoclonal antibodies used were flavivirus genus 4G2, dengue complex 2H2[14] and yellow fever specific 2D12.[15] The MAF and monoclonal antibodies were used in the IFA at a 1:50 and/or 1:100 dilution.

### Management of cases

Immediately after the first cases were reported two laboratory teams, with basic equipment to perform blood and urine analysis, were transported by helicopter and installed in the outbreak foci. The “jungle laboratories” became the key elements in the referral of severe cases to Internal Medicine and Intensive Care Unit of the Armed Forces Hospital No.1 located in Quito at 2.800 meter above the sea level, where no vectors of yellow fever exists, to avoid the possible risk of initiating an urban cycle in Puyo and/or Guayaquil lowlands cities where the closest military hospitals were located. Symptomatic cases were closely monitored in two jungle health posts; those patients who showed an increase in SGOT or SGPT or albumin in urine were evacuated by helicopter to the Armed Forces Hospital No 1 in Quito.

### Statistical analysis

Log-linear regression analysis was used to evaluate age and presence of antibodies against dengue type2 virus as predictors of yellow fever infection or severe disease. Yellow fever cases were defined as symptomatic subjects with positive IgM, those with IgG greater than 1:200; or those from whose serum yellow fever virus was isolated. Severe yellow fever cases were defined as those who entered in the period of intoxication with presence of jaundice, albuminuria, hematuria, blood in feces, or a combination of these symptoms. Relative Risk (RR) and 95% confidence intervals (CI) were calculated. Log-linear models were chosen to avoid hyperinflation of the parameter estimates when using logistic regression models.

## RESULTS

During the outbreak investigation, 44 yellow fever cases and three casualties were reported among 341 subjects who had not been immunized prior to their detachment in the Amazonian rainforest. An estimated attack rate of 13% (44/341) with a case fatality rate of 6.8% (3/44) was observed. This study is restricted to the 338 survivors, being 97% of the active personnel of the garrison. Most of subjects were recruits assigned to one year of jungle training. Of these, 174 were from the Coastal area of the country, 73 from the Andean zone of the country, and 91 were natives of the Amazonian rainforest. The onset of the first case was July 1, 1997, and the onset of the last documented case was on August 5, 1997. The outbreak dissemination was rapidly controlled by a 100% immunization of the study subject after the collection of blood samples. Three acute cases of Mayaro virus infection, three acute cases of Oropouche virus infection and one case of HIV infection was detected during investigation. *Plasmodium* in blood was detected in two cases and three controls, there were four with *P. vivax* and one patient with both *P. vivax* and *P. falciparum*.

During the seroepidemiological survey, the presence of dengue antigens among yellow fever cases was observed at the following rates: 17 out of 22 (77.3%) among personnel from the coastal region, one in seven (14.3%) of the personnel from the Amazon and two of 12 (16.7 %) in personnel from the Andean region.

After controlling by risk factors described in the tables, the presence of dengue antibodies (RR=1.5; CI=0.9-2.4) was not significantly associated with yellow fever infection [Table 1], but it was significantly associated with severity of yellow fever cases (RR=0.2; CI=0.1-0.9) [Table 2].

**Table 1:** Dengue and yellow fever infection

Risk factor	Yellow fever		Crude attack rate %	RR^1^	CI	*P* value
					
	Positive	Negative				
Antibodies against dengue						
Yes	20	102	16	1.5	0.9-2.4	0.1110
No	21	195	10	1.0	(ref)	
Age						
≤19 years	20	172	10	0.9	0.5-1.5	0.6198
>19 years	21	125	14	1.0	(ref)	
Clearing the rainforest						
Yes	36	181	17	5.2	2.1-13.3	0.0005
No	5	116	4	1.0	(ref)	
Localization						
Detachment or outpost	21	49	30	4.8	2.3-10.2	0.0001
Garrison	20	248	8		(ref)	
Use of bed net						
Yes	38	270	12	0.6	0.2-2.2	0.4484
No	3	27	10		(ref)	
Use of repellent						
Yes	22	241	8	0.7	0.4-1.5	0.4170
No	19	56	25		(ref)

Relative Risk (RR) and 95% confidence interval (CI) of the RR, from a log-linear regression model including terms for all variables in the table.

**Table 2:** Dengue and severe yellow fever

Risk factor	Severe yellow fever cases		Severity rate %	RR^*^	CI	*P* value
					
	Yes	No				
Antibodies against dengue						
Yes	2	18	10	0.2	0.1-0.9	0.0352
No	9	12	43	1.0	(ref)
Age						
≤19 years	3	17	15	0.4	0.1-1.3	0.1223
>19 years	8	13	38	1.0	(ref)

* Relative Risk (RR) and 95% confidence interval (CI) of the RR, from a log-linear regression model including terms for all variables in the table.

## DISCUSSION

Antibodies against dengue were observed in a high percentage of military personnel from the coastal region, where dengue is considered an endemic disease. Dengue is one of the main causes of arthropod-borne viral illnesses in the Ecuadorian coast. In a serologic survey carried out in 1988 in the port of Guayaquil, 422,000 of the approximately 1.2 million inhabitants were found to have been previously infected with dengue.[16]

This study did not associate dengue antibodies with yellow fever infection but with a less severe clinical presentation. In 1970, Theiler and Anderson reported the experimental challenge with the JSS South American yellow fever virus in 2 monkeys previously immunized with pooled human sera from volunteers infected by Dr Albert B Sabin with dengue type 1 (Hawaii) virus, and two non-immunized control monkeys. After challenge with the JSS fever virus, the control monkeys circulated significantly more viruses than did dengue-immune monkeys. The titers of circulating virus were six per 0.03 ml in the control monkeys and 4.5 per 0.03 ml in the dengue-immune monkeys.[17] An experiment with a hamster model of the disease to study immunologic cross-protection by other flaviviruses against yellow fever, showed a delayed in virus replication and lower peak titers in hamsters previously exposed to other flavivirus compared to naive hamsters.[7] Gomez and Ocazionez (2008) also reported similar results after observing that sera from individuals with dengue antibodies, no previously vaccinated against yellow fever, neutralized the yellow fever 17D virus. In addition, the dengue immune sera significantly (*P* less than 0.001) neutralized the yellow fever 17 D virus when compared with non-dengue immune sera.[18].

The findings of our study suggest that previous exposure to dengue virus in humans may reduce yellow fever viremia and ameliorate the severity of the disease, reducing the fatality rate without decreasing the rate of infection. None death was reported among individuals from the coastal region (22 individuals), while a case fatality rate of 14% (3/22) was registered among individuals from the Andean and Amazon region. The protective value of previous exposure to flavivirus against yellow fever mortality was evidenced in the epidemiological observations done in the 10^th^ Regiment of Infantry of the Napoleonic forces. In 1815, during the Napoleonic wars, Pym reported that only four of 408 officers/men who contracted yellow fever in Gibraltar, after service in India, died. From this information, the fatality rate for that group was as low as 0.98%. On the other hand, among 55 men who contracted yellow fever but had not been in India, 21 deaths were observed. Among those 55 men, the fatality rate for yellow fever was as high as 38.18%. Based on this information, Pym recommended that the troops should be “seasoned” in the East Indies before being detached to the West Indies.[19]

Yellow fever virus wild type and vaccine strains induce a broad heterotypic serological response in individuals with preexisting flavivirus antibodies.[20,21] In 1984, Monath showed that yellow fever virus possesses antigenic determinants that are common in several flavivirus groups, and proposed the theory of the “original antigenic sin”. This phenomenon has been observed when inoculation with the 17D yellow fever vaccine have produced a homotypic in immune virgin individuals, or a broad and heterotypic response in individuals who have had previous flavivirus contact.[22]

Protective cross-reaction may act by both antibody and cellular immune response. It has been established that all flavivirus share common antigenic determinants generally related to the E protein, which is the target for both the hemaglutination inhibition assay and the neutralization test. Because of its role as the major virion surface component, the E protein plays an outstanding role in the generation of neutralizing antibodies and in the induction of a protective immunity.[6] At the cellular level, memory T lymphocytes induced by primary flavivirus infections can be activated during secondary flavivirus infections, and may modulate the outcome of the secondary infection.[5] A primary infection with one serotype dengue virus induces virus-specific CD4+ and CD8+ memory T lymphocytes.[23] Concomitantly, flavivirus-cross-reactive memory T cells have been detected in some individuals experimentally immunized with a dengue type 2 vaccine.[24,25]

The omission of possible predictor variables such as genetic factors or virulence of the yellow fever strain is a limitation and should be considered in interpreting the results. Nevertheless, having wholly captive study population makes this yellow fever outbreak research unique, and the analytical technique used provides conservative values for the parameters estimated.

## CONCLUSIONS

During the present yellow fever outbreak, a high percentage of cases among military personnel from the coastal area, where dengue is endemic, showed antibodies against dengue. The anamnestic dengue response observed confirms the broad heterotypic flavivirus response reported in previous studies. The findings of this study suggest that previous exposure to dengue infection may have determined a secondary immune response that did not decrease the rate of infection but ameliorated the course of yellow fever infection.
